# MicroRNAs and RNA-Binding Protein-Based Regulation of Bone Metastasis from Hepatobiliary Cancers and Potential Therapeutic Strategies

**DOI:** 10.3390/cells13231935

**Published:** 2024-11-21

**Authors:** Sharmila Fagoonee, Ralf Weiskirchen

**Affiliations:** 1 Institute of Biostructure and Bioimaging (CNR), Molecular Biotechnology Center “Guido Tarone”, 10126 Turin, Italy; 2 Institute of Molecular Pathobiochemistry, Experimental Gene Therapy and Clinical Chemistry (IFMPEGKC), RWTH University Hospital Aachen, D-52074 Aachen, Germany

**Keywords:** hepatocellular carcinoma, cholangiocarcinoma, bone metastases, extracellular vesicles, RNA-binding proteins, microRNAs, therapy, biomarkers

## Abstract

Hepatobiliary cancers, such as hepatocellular carcinoma (HCC) and cholangiocarcinoma (CCA), are among the deadliest malignancies worldwide, leading to a significant number of cancer-related deaths. While bone metastases from these cancers are rare, they are highly aggressive and linked to poor prognosis. This review focuses on RNA-based molecular mechanisms that contribute to bone metastasis from hepatobiliary cancers. Specifically, the role of two key factors, microRNAs (miRNAs) and RNA-binding proteins (RBPs), which have not been extensively studied in the context of HCC and CCA, is discussed. These molecules often exhibit abnormal expression in hepatobiliary tumors, influencing cancer cell spread and metastasis by disrupting bone homeostasis, thereby aiding tumor cell migration and survival in the bone microenvironment. This review also discusses potential therapeutic strategies targeting these RNA-based pathways to reduce bone metastasis and improve patient outcomes. Further research is crucial for developing effective miRNA- and RBP-based diagnostic and prognostic biomarkers and treatments to prevent bone metastases in hepatobiliary cancers.

## 1. Introduction

Hepatobiliary cancers, including hepatocellular carcinoma (HCC), intrahepatic cholangiocarcinoma (iCCA), gall bladder cancer (GC), extrahepatic cholangiocarcinoma (eCCA), and ampullary cancer, are among the deadliest malignancies and account for approximately 13% of cancer-related deaths worldwide [1]. HCC develops in chronic liver inflammatory conditions, such as viral infections like hepatitis B virus (HBV) or hepatitis C virus (HCV), metabolic dysfunction-associated steatohepatitis (MASH), alcohol consumption, tobacco smoking, primary biliary cholangitis, and hemochromatosis, with the molecular pathogenesis varying based on the genotoxic insults and etiology [2,3]. Unresolved fibrosis from any etiology leading to cirrhotic changes is the primary risk factor for HCC. CCA arises against a background of biliary inflammation and fibrosis, originating from biliary epithelial cells or cholangiocytes lining the intrahepatic bile duct [4]. Among biliary tract cancers, iCCA (discussed in this review as representative of CCA) can also develop from chronic viral infections (HBV or HCV) or MASH, as well as biliary tract disorders, like choledochal cyst, cholelithiasis, primary sclerosing cholangitis (PSC), environmental toxins, and cirrhosis [5]. The pathogenesis of iCCA is complex and involves genetic mutations in key tumor suppressors, such as *TP53*, or in oncogenes like *K-RAS* [6]. Several effective treatment options exist for HCC, including hepatectomy, radiofrequency ablation, or chemotherapy with tyrosine kinase inhibitors, such as sorafenib [7]. Liver transplantation has also emerged as a promising treatment option, where feasible, for these malignant hepatobiliary diseases. However, given the high number of deaths from these malignant liver diseases, the search for alternative therapeutic strategies is ongoing. Multiple molecular pathways are impacted by HCC and iCCA (discussed below) and are the focus of studies on novel therapeutics or diagnostic/prognostic biomarker development.

Metastasis occurs in 11.2–25.5% of patients affected by HCC and in 42.62% of those affected by iCCA [8,9]. The most common sites for HCC metastasis are the lungs, portal vein, and portal lymph nodes, while iCCA primarily metastasizes to the lungs, peritoneum, and lymph nodes [10,11]. Patients with metastasis from these cancers have a poor prognosis. Importantly, distant metastasis to the bones and brain also confers a poor outcome to HCC and iCCA patients at advanced stages of the diseases. Several studies have investigated the importance of the metastatic site on patient survival. Brain metastasis is rare for both HCC and iCCA (around 2% for HCC and around 0.15% for CCA), while bone metastasis is more common. Patients with bone metastasis tend to have a worse prognosis compared to those with liver metastases [12,13,14]. Bone metastases enhance morbidity and mortality and usually occur in the axial skeleton, where they exhibit common patterns of lesions [15]. The latter are classified as follows in relation to the primary mechanism of hindrance of normal bone remodeling: (i) osteolytic (destruction of normal bone); (ii) osteoblastic (or sclerotic; deposition of new bone); (iii) mixed, if both osteolytic and osteoblastic lesions occur [16]. Bone metastases from HCC are mostly osteolytic, while those from CCA show a mixed osteolytic/osteosclerotic pattern [17,18]. In this review, we aim to provide an update on the role of RNA-based mechanisms, such as microRNAs (miRNAs) and RNA-binding proteins (RBPs), in bone metastasis from hepatobiliary cancers (HCC and CCA). We also discuss how this knowledge can be applied to develop new therapeutic strategies and biomarkers to target this life-threatening complication of primary cancers.

## 2. Metastasis from Hepatobiliary Cancers

### 2.1. Impact on Prognosis and Survival of Patients

#### 2.1.1. Hepatocellular Carcinoma (HCC)

Bone metastases from HCC are highly vascularized and often result in severe pain, as well as significant structural and functional damage, including pathologic fractures and neurological deficits. These complications greatly reduce patients’ quality of life [19]. Radiation therapy is a widely accepted treatment for metastatic bone lesions, as it provides relief for painful areas. Pain relief after radiation therapy is achieved in approximately 60% to 95% of cases, with complete pain resolution in up to 32%. Pathologic fractures and neurological complications can also occur.

Advances in the detection of bone metastasis from HCC have revealed that around 16.1% to 38.5% of HCC patients present with bone metastasis at the time of diagnosis, and 11.7% develop bone metastases after undergoing curative resection [20]. In a study from China, the most common site for bone metastasis was found to be the trunk (approximately 69%), followed by the lower limb (19%), upper limb (9%), and skull (3%). These patients generally have poor prognoses, with a median survival of 11 months following the diagnosis of bone metastasis (range: 4 to 52 months). The 1-year survival rate was 44.2%, while the 2-year survival rate dropped to 11.6% [21].

In another study, the survival rates decreased from 35.5% to 13.5% during the first and second year, respectively [19]. The median survival time was reported to be from 2 to 4.6 months [22]. Worse overall survival among HCC-bone metastasis patients was associated with Child–Pugh class A group, alpha-fetoprotein (AFP) levels over 30 ng/mL, a tumor size greater than 5 cm, and skeletal-related events [19].

Despite the grim prognosis for HCC patients with bone metastasis, early detection is crucial for timely intervention and appropriate treatment. This underscores the importance of screening programs for detecting HCC early to prevent metastatic spread at advanced stages [23]. Once metastasis occurs, curative treatment options, such as surgical resection, liver transplantation, or local ablative therapies, are no longer viable or effective.

#### 2.1.2. Cholangiocarcinoma (CCA)

A recent pan-European observational study reported that iCCA preferentially metastasizes to the lungs (32.9%), distant lymph nodes (30.5%), peritoneum (26.4%), and bone (13.8%) compared to perihilar or distal CCA, which mainly metastasize to the liver or peritoneum [24]. Another study conducted on 186 metastatic iCCA patients showed that 104 had metastasis to the liver only, while 20 (11%) showed metastasis to the bone. The latter was more common in males compared to females (11 versus 9) [11]. Like HCC, several studies have explored the impact of the metastatic site on survival in CCA. It was found that bone metastases in CCA patients led to an exceptionally poor prognosis, with survival averaging around 4 months [11]. Bone metastases are frequently located in the humerus, fibula, and femur, with few reported cases of metastasis to the appendicular skeleton from CCA [25]. Imaging modalities, such as positron emission tomography (PET)/computed tomography (CT) and PET/magnetic resonance imaging (MRI), have shown high sensitivity and specificity in detecting skeletal metastasis, but they are not routinely used in patients initially diagnosed with CCA [25]. Bone metastases are typically identified when bone-related symptoms, like pain and pathological fractures, manifest. The prognosis remains bleak, as traditional chemotherapy and radiotherapy yield minimal responses. Palliative care, primarily focusing on symptom management, especially pain relief, remains the mainstay of treatment aimed at improving patients’ quality of life.

### 2.2. Hepatobiliary Metastatic Modifications and Dissemination of Cancer Cells

The biological mechanisms underlying the shedding of tumor cells from HCC and CCA and their dissemination from primary tumor sites to other organs are similar to those of other malignant tumors and have been extensively described elsewhere [26,27]. Briefly, the complex interplay among several factors, including genetic instability, cellular transformation, epigenetic alterations, and host immune response, leads to aberrations in cell signaling pathways. Dysregulation in cellular proliferation, unresponsiveness to growth inhibitory mechanisms, escape from apoptosis, epithelial to mesenchymal transition (EMT), enhanced tumor–stromal interactions, and angiogenesis are the main factors. Neo-angiogenesis is initiated to provide oxygen, nutrients, and growth factors to tumor cells, facilitating their growth and invasion. Vascular endothelial growth factor (VEGF), a key regulator of angiogenesis in both primary HCC and its metastasis to bone, plays a crucial role in promoting bone resorption and enabling tumor proliferation within the bone [28]. Iguchi et al. demonstrated that VEGF levels are significantly higher in HCC patients with bone metastasis compared to those without bone metastasis or only with chronic hepatitis/cirrhosis [28]. Following vascular invasion, metastatic cancer cells, known as circulating tumor cells (CTCs), can be identified in the bloodstream or lymphatic system. The detection of CTCs or circulating DNA derived from tumor cells (ctDNA) of HCC is linked to a more aggressive disease phenotype and a worse prognosis [29]. HCC cells that infiltrate the bone marrow space can secrete VEGF, promoting osteolytic bone metastasis. Furthermore, once bone invasion is established, VEGF can enhance tumor growth in this environment by acting as an angiogenic factor. Mesenchymal-to-epithelial transition (MET) is also a critical process that occurs at metastatic sites, influencing the successful growth of tumor cells after homing. Moreover, dysregulation in the post-transcriptional control of gene expression mediated by various non-coding RNAs (ncRNAs) or RBPs significantly impacts tumorigenesis and is discussed further below. In the pathogenesis of HCC and CCA, modulation of non-coding RNAs and RBPs can also trigger the malignant transformation of hepatobiliary cells.

### 2.3. RNA Biology in Metastatic Cancers

Complex regulatory mechanisms ensure that the correct information is fed into each cellular pathway at the right quantity, time, and place. RNA, with a plethora of functions and properties, can exert this information-providing regulatory role, not only as mRNA but also as other non-coding RNA species, including miRNAs, long ncRNA (lncRNA), circular RNA (circRNA), and PIWI-interacting RNA (piRNA), all potentially involved in cellular physiology and pathology [30]. This complex scenario is further complicated by processes like alternative splicing, RNA editing, RNA modification, and RNA folding, making the RNA world one of the most explored avenues in modern oncology [31]. RNA species can act as catalysts of enzymatic reactions, tools for silencing gene expression, and, due to their differential expression, also as biomarkers in human pathologies. Recently, due to the advent of high through-put sequencing technologies and the capacity to bioinformatically deal with big data, increasing knowledge has been acquired regarding the role of RNA in malignant transformation. The function of lncRNAs, for example, is now emerging in the context of hepatobiliary tumors [32]. Two lncRNAs, HULC and H19, showed enhanced expression in CCA cells and acted as competing endogenous RNAs (ceRNAs) or sponges for let-7a/let-7b and miR-372/miR-373, involved in the IL6/CXCR4 inflammatory pathway to control cell migration and invasion [33]. HULC is also overexpressed in HCC tissues and was found to enhance HCC cell proliferation and metastatic potential through the miR-2052/MET receptor tyrosine kinase axis in vitro and in vivo [34]. Moreover, H19 is also overexpressed in cancer stem cells in HCC and is promising as a biomarker for this tumor [35]. However, another study found decreased expression of H19 in the tumor tissue of HCC patients versus normal or non-tumorous adjacent tissue, and overexpression of H19 could suppress tumor growth and increase chemosensitivity [36]. Importantly, the role of lncRNAs in bone metastasis is also emerging. H19, for instance, is overexpressed in HCC patients with a high bone metastasis tendency and was found to promote p38MAPK dephosphorylation by facilitating the interaction between protein phosphatase 1 catalytic subunit alpha (PPP1CA) and p38 in preclinical studies [37]. This led to reduced osteoprotegerin (OPG) expression, which, when coupled to RANKL in a specific ratio, is capable of inhibiting osteoclastogenesis and bone metastasis. H19 also sponged miR-200b-3p, which targets zinc finger E-box binding homeobox 1 (Zeb1), an EMT inducer. Overall, these studies point out how standardization of analytical instruments and tools, as well as patient stratification according to their tumor molecular profile are required before these RNA species can become part of the routine diagnosis and therapeutic regimen.

#### 2.3.1. Alternative Splicing

Constitutive RNA splicing, a process in which introns are removed from mRNA precursors to connect exons and create a single type of mRNA from a single gene, has been extensively described in [38]. On the other hand, alternative splicing results in the generation of RNA variants through the selection of alternative 5′ or 3′ splice sites, increasing transcriptome complexity and playing a role in physiological and pathological processes in human diseases (as extensively described in [39,40]). Spliceosomes, large ribonucleoprotein (RNP) complexes composed of small nuclear RNAs (snRNAs U1, U2, U4, U5, and U6) and various splicing protein factors/RBPs, recognize and bind consensus sequences located at the 5′ and 3′ ends of each intron [41]. This is followed by two consecutive transesterification reactions, which result in the removal of the intron (or skipping of several exons along with introns) and the covalent joining of the remaining adjacent exons [39].

Strong or weak splice sites determine the amount of mature mRNA variants that are generated. The choice of splice sites is regulated by cis- (intrinsic sequences) and trans- elements (splicing factors), as well as by Pol II in a complex regulatory mechanism involving chromatin conformation. Splicing factors recognize regulatory sequences (exonic splicing enhancers and silencers (ESEs and ESSs), as well as intronic splicing enhancers and silencers (ISEs and ISSs)) located on introns or exons. The spliceosome acts through four stages (assembly, activation, catalysis, and disassembly), which are tightly regulated. Dysregulation in any of these stages can lead to pathological alterations in the cell and cancer. In fact, alternative splicing events have been associated with the regulation of genes and pathways, leading to the development of HCC and CCA. For instance, several splicing factors show expression dysregulation in HCC (including *ESRP2*, *HNRNPAB*, *PCBP2*, *SFPQ*, *RBM8A*, *RBMS1*, *MATR3*, and *ZCRB1*) and in CCA (including *RBM10* and *METTL14*) and impact proliferation, epithelial–mesenchymal transition (EMT), drug resistance, etc. [42,43]. Alternatively spliced transcripts also affect tumor progression. CD44 is one example of this. CD44 pre-mRNA can be alternatively spliced into CD44 variable (v) or standard (s) isoforms, for instance, by the RBP epithelial regulatory splicing factor 1 (ESRP1), resulting in the generation of transcripts with a variable number of exons between exon 5 and 16 or the complete lack of exons between exon 5 and 16, respectively [44]. In HCC, the CD44s isoform is elevated and has been shown to promote caspase1/interleukin (IL)-1β activation, thereby contributing to HCC metastasis and resistance to chemotherapy [45]. In CCA, CD44s expression is necessary for the invasive phenotype of tumor cells, and the silencing of CD44s leads to a reduction in migration and adhesion of CCA cells in vitro. Interestingly, Nanashima et al. reported that 18% of iCCA patients had their tumor sections stain positive for CD44, while Pongcharoen et al. showed that CD44 was mainly expressed on the plasma membrane of cells at the invasive front areas of CCA, with a significant correlation with poorly differentiated mass-forming type CCA [46,47]. Thus, alternatively spliced transcripts and differential expression of spliceosomal components can develop into useful biomarkers and therapeutic targets.

#### 2.3.2. mRNA Translation Control

RNA chemical modifications have long been known to occur post-transcriptionally in response to cellular and extracellular stimuli and to increase the functionality of RNA species [48]. Such modifications can affect RNA transcription, stability, and translation, as well as localization. For instance, methylation of RNA is a common post-transcriptional alteration critically affecting RNA function and cellular homeostasis. N6-methyladenine (m6A)-modified transcripts are bound by m6A RNA-binding proteins, the YTH domain family (YTHDF). YTHDF2 was found to be overexpressed in HCC and associated with tumor cell proliferation and could be targeted by miR-145 [49]. Sublethal heat stress resulting from insufficient radiofrequency ablation in HCC was found to induce the m6A modification of EGFR transcripts, leading to their binding by YTHDF1 and resulting in enhanced HCC cell survival [50]. m1A, m5C, and m7G RNA modifications are also important in HCC [51].

Factors that affect mRNA translation can alter cell fate, including survival, proliferation, differentiation, and death. Cellular stress can reshape the proteome through various processes, such as chromatin remodeling, mRNA production, translation, mRNA decay, protein modification, and protein degradation [52]. Different pathways regulate mRNA translation, particularly in response to stress. Depending on whether the damage is reversible or not, the activation of these pathways can either lead to cell survival or death. One such pathway involves the phosphorylation of the eukaryotic Initiation Factor 2α (eIF2α) subunit of eIF2, a eukaryotic initiation factor responsible for recognizing the AUG start codon on initiator Met-tRNA and forming the 43S pre-initiation complex. This can repress cellular mRNA translation rates and protein synthesis. Several eIF2α kinases exist and are specifically recruited to phosphorylate eIF2α based on the stimulus received [52].

For example, the general control nonderepressible 2 (GCN2)/EIF2AK4 kinase modifies eIF2α under nutritional and UV stresses. Interestingly, this kinase is also implicated in tumorigenesis and can regulate amino acid transporter solute carrier (SLC) gene expression through ATF4 activation in MYC-overexpressing cancer cells [53]. The activation of the GCN2–eIF2α–ATF4 axis can promote VEGF expression and angiogenesis in response to amino acid deprivation, facilitating the angiogenic switch necessary for tumors to re-establish their blood supply following nutrient and oxygen deprivation [53].

It has been demonstrated that inhibiting GCN2 in arginine-deprived HCC cells sensitizes them to senotherapy, resulting in increased senescence in vitro [54]. Tumor regression was also observed in vivo in xenograft tumor models (SNU-398 HCC cell-inoculated mice) that were fed an arginine-free diet and treated with a GCN2 inhibitor and senolytic compounds (such as ABT-263, a Bcl-2/Bcl-xl inhibitor) compared to control groups. Understanding RNA modifications and translational control mechanisms is crucial in developing new therapies for HCC and CCA.

## 3. Dysregulated Expression of miRNAs and RBPs as Potential Biomarkers of Bone Metastasis from Hepatobiliary Tumors

The introduction of modern treatments, such as immunotherapies, in recent years has drastically changed the management of HCC and CCA. These changes require more monitoring, preferably through biomarkers. Biomarkers have become valuable tools for diagnosing, predicting treatment responses, and improving patient selection. However, overall survival remains poor, highlighting the need for better strategies to improve patient outcomes. The search for cancer biomarkers is an actively evolving field. In addition to searching for dysregulated serum proteins, like AFP or glypican-3 (GPC3) for HCC, and carbohydrate antigen 19-9 (CA19-9) for CCA, biomolecules involved in RNA metabolism, such as miRNAs and RBPs, have also attracted attention [55,56,57].

### 3.1. Dysregulated miRNA Expression

Small non-coding RNAs (ncRNAs) are a diverse group of gene expression regulators that include molecules less than 200 nucleotides long, such as miRNAs and small interfering RNAs. The role of these molecules in modulating carcinogenesis is constantly being investigated [58]. MiRNAs are RNA molecules that are 19–25 nucleotides long and play a role in mRNA degradation and translational repression, ultimately controlling protein turnover [59]. It is estimated that miRNAs regulate around 30% of human genes, many of which are involved in the production and pathways of carcinogenic molecules [60]. These biomolecules can be seen as a double-edged sword due to their substantial involvement in carcinogenesis, either acting as tumor suppressors or as oncomiRs. Dysregulated expression of miRNAs is closely linked to changes in target expression that occur during tumorigenesis. In fact, an in-depth molecular analysis of HCC and CCA samples versus controls has highlighted the dysregulated expression of several miRNAs in hepatobiliary tumors (Table 1).

miRNA signatures obtained through miRNA profiling of tumors versus “healthy” tissue can provide insights into malignant tumor diagnosis, staging, prognosis, and response to anti-tumor therapies. For example, a survival estimation method based on miRNA signatures from 122 HCC patients showed that 32 miRNAs could predict survival time in these subjects [79]. Interestingly, seven prognostic indicators (miR-146a-3p, miR-200a-3p, miR-652-3p, miR-34a-3p, miR-132-5p, miR-1301-3p, and miR-374b-3p) and four diagnostic indicators (miR-1301-3p, miR-17-5p, miR-34a-3p, and miR-200a-3p) were identified. Out of these, three miRNAs (miR-200a-3p, miR-1301-3p, and miR-17-5p) also showed a tumor-stagewise trend. These data are promising and warrant further clinical investigation.

### 3.2. Dysregulated RBP Expression

**Figure 1:**
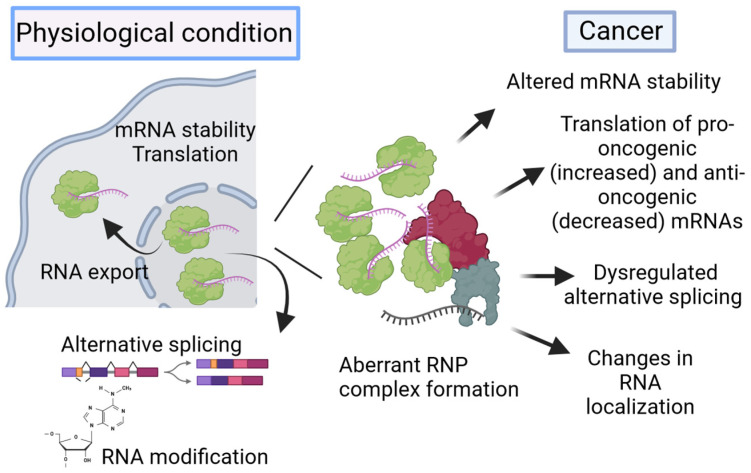
Most common functions of RBPs under physiological conditions or in cancer. In healthy cells, RBPs can post-transcriptionally regulate RNA metabolism, including alternative splicing, RNA modification, mRNA stability, and translation. This is represented in a simplified version in the figure. Under pathological conditions, increased expression of RBPs can lead to aberrant RNP formation through recruitment of oncogenic proteins, RBPs, and diverse RNA species, leading to dysregulated mRNA stability, translation, and alternative splicing, as well as alterations in cellular localizations of RNAs.

A second level of control comes from RBPs, which are also involved in RNA metabolism and modulate RNA stability, alternative splicing, translation, modification, and localization. Importantly, numerous RBPs interact with multiple RNA species through one or multiple globular RNA-binding domains, including messenger RNA (mRNA), miRNA, lncRNA, and circRNA [80]. Precise regulation of RBP expression is essential for maintaining cellular homeostasis due to the RPBs’ participation in the maintenance of balanced cell physiology and homeostasis. Any perturbation in the expression of RBPs or activity may be responsible for aberrant RNP complex formation, and hence, participate in pathological processes, such as in cancer [81]. Some hints regarding how the different functions of RBPs affect tumorigenesis are shown in Figure 1.

Moreover, the regulators of RBPs and other spliceosome components, such as serine-arginine (SR) RBPs and splicing kinases, including CDC-like kinases, SRSF protein kinases, and pre-mRNA splicing 4 kinase, are dysregulated in cancer [82]. RBPs are considered key modulators of various pathological processes, including cell proliferation, invasion, and metastasis, in HCC and CCA, suggesting RBPs as potential therapeutic targets for treating these tumors. An integrated analysis of RBPs from The Cancer Genome Atlas (TCGA) HCC datasets revealed that 330 RBPs were differentially expressed. These RBPs were used to create an 8-RBPs signature (*SNRPD1*, *IARS*, *BRCA1*, *EZH2*, *RUVBL1*, *TST*, *TCOF1*, and *AZGP1*) with high prognostic potential for clinical therapeutic intervention in this cancer [83]. RBPs also play a crucial role in bile acid homeostasis and biliary metabolism, and their dysregulated expression can contribute to cholangiocarcinogenesis. In the context of HCC and CCA, some RBPs have been studied for their involvement in cancer progression and metastasis. However, research specifically linking RBPs to bone metastasis is still emerging. Although bone metastasis is less common in HCC and CCA compared to liver metastasis, some RBPs involved in these cancers may influence metastatic potential and promote bone metastasis in some cases. Abnormal RBP expression has been reported in several studies on HCC, while RBP expression analysis remains understudied in CCA (Table 2).

Although no specific RBP has been definitively identified as a biomarker of bone metastasis in HCC or CCA, RBPs such as IGF2BP1, HuR, LIN28, and FXR1 are known to be involved in promoting general metastasis and aggressive tumor behavior [95,96,97,98]. These proteins may play a role in the process of bone metastasis, particularly by influencing EMT, cancer cell migration, and invasion. Further research is necessary to determine specific RBPs as biomarkers for bone metastasis in these cancers.

As “prevention is better than cure”, it is important to focus research efforts on blocking the metastatic cascade in the primary tumor. Thus, finding biomarkers for each step preceding metastasis is essential. Recently, we examined gene expression repositories to generate a list of differentially expressed RBPs in PSC predicting its progression to CCA [80]. This study revealed an increase in the level of RBP transcripts, *FANCD2* and *ASPM*, in the body fluids of PSC and CCA patients compared to their respective controls. This reflects the expression trends in the tissues, suggesting that this approach can uncover potential biomarkers for non-invasively predicting the evolution to metastatic tumors. In an attempt to create and validate an RBP-related model for predicting the prognosis of HCC patients, Tian et al. bioinformatically analyzed differentially regulated RBP transcripts in HCC versus adjacent non-cancer tissue [99]. A prognostic model was developed based on two RBPs, allowing for risk-stratification of individuals with HCC. Specifically, block of proliferation (*BOP1*) was found to be overexpressed in HCC tissues compared to non-tumor tissue, showing a strong association with microvascular invasion and an advanced pathological stage [99]. Additionally, *EZH2* overexpressed in HCC, was significantly correlated with tumor grades and prognosis in HCC patients. Insights can also be gained from other tumors that more commonly metastasize to the bone compared to HCC and CCA, such as breast cancer. In addition to α-fetoprotein, carcinoembryonic antigen, prostate-specific antigen, cytokines, and chemokines, miRNAs like miR-214-3p, -218, -126, -206, and -335 have been shown to play a crucial role in bone metastasis development, particularly in preclinical models [100,101]. Elevated miR-21 and miR-141 have been observed in breast cancer and prostate cancer patients with a predisposition for bone metastasis, respectively [101,102]. A similar approach can be taken to identify biomolecules for early detection of HCC and CCA patients at high risk for bone metastasis.

### 3.3. RBP/miRNA Crosstalk

miRNAs and RBPs, as key players in post-transcriptional regulation of gene expression, can co-operate, antagonize, or regulate each other. RBPs and ribonucleases tightly regulate the biogenesis of miRNAs at both the transcriptional and post-transcriptional levels, as well as their turnover, while miRNAs can target RBPs and decrease their expression. For example, the RBP heterogeneous nuclear ribonucleoprotein D (hnRNPD), also known as AU-rich element RNA-binding protein 1 (AUF1), can modulate miR-122 expression in HCC by affecting the processing of pre-miRNA into mature miR-122. Mechanistically, hnRNPD inhibits Dicer expression by interacting with the 3′UTR and open reading frame (ORF) of *Dicer1* mRNA in HCC cells [103,104]. Splice factors, such as SRSF1, SRSF2, and hnRNP A1, are targeted by miRNAs like miR-10b-5p, miR-203a-3p (SRSF1), miR-183-5p, miR-200c-3p (SRSF2), miR-135a-5p, and miR-149-5p (hnRNP A1) in oncogenesis [105]. RBPs play a role in regulating miRNA expression in cancer, as extensively described elsewhere [104]. Conversely, RBPs have potential binding sites for several miRNAs on their 3′UTR. For instance, the RBP cytoplasmic polyadenylation element-binding protein 4 (*CPEB4*) is targeted by miR-550a in HCC, leading to increased migration and invasion of HCC cells in vitro [106,107]. A deeper understanding of the complex reciprocal regulation between miRNAs and RBPs is needed, as it may offer additional therapeutic targets in the field of hepatobiliary diseases and bone metastasis.

## 4. Molecular and Cellular Alterations Leading to Bone Metastasis from Hepatobiliary Cancers and Indicative Therapeutic Targets

An important issue under investigation is what pathological processes drive a small proportion of HCC and CCA cells (usually around 0.02–<1%) to colonize the bone microenvironment, and what remodeling activities occur in the bone marrow to facilitate this invasion by tumor cells [108,109]. Understanding the molecular mechanisms that explain the osteotropism of metastatic cells to the bone micro-environment and how they survive and thrive in the bone marrow and grow into macro-metastasis is crucial in the search for new therapeutic strategies to reduce metastasis. Cells disseminating from primary tumors of the breast, lung, prostate, thyroid, and kidney, for instance, find fertile soil in the bone marrow, created by survival- and growth-promoting factors released by osteoclasts, osteoblasts, and bone stromal cells, which explains the frequent bone metastasis occurring in patients affected by these malignancies [16]. The bone metastatic niche is also influenced by the vasculature system that supports both osteogenesis and angiogenesis and the osteocytes’ response to environmental and mechanical stimuli and signals, as well as the contribution of immune cells and metastasis-associated fibroblasts [108,110]. There are reports on pre-conditioning of the microenvironment starting long before the journey of the metastasizing cells from the primary tumor site to a predetermined location, such as the bone. For instance, vascular VEGF-A is secreted by primary tumors to promote angiogenesis, mobilize hematopoietic bone marrow progenitor cells, and immunomodulate the metastatic niche prior to the arrival of the cancer cells [111,112].

Interestingly, there are eight splicing isoforms of VEGF-A, some with pro-angiogenic activities (VEGF-A_xxx_a) and others with anti-angiogenic properties (VEGF-A_xxx_b). The VEGF-A_xxx_b isoforms are less effective in activating the VEGFRs. RBPs, such as SRSF1 and heterogenous nuclear ribonucleoprotein L (hnRNP L), and miRNAs, such as miR-297, miR-299, and miR-574-3p, participate in the regulation of *VEGF-A* splicing and expression, respectively [113,114]. Circular RNAs, like circSMARCA5, also play a role in regulating *VEGF-A* mRNA splicing by acting as a sponge for SRSF1, thereby dampening its activity and the synthesis of VEGF-A_xxx_a isoforms [115]. It is important to investigate the complex interaction of post-transcriptional regulators in HCC and CCA angiogenesis to identify new molecular targets and biomarkers. Other mediators derived from primary tumors include cytokines, chemokines, growth factors, free RNAs, metabolites, and biomolecules derived from extracellular vesicles (EV) [116]. Cancer-associated fibroblasts (CAFs) also promote HCC metastasis through chemokine-activated hedgehog and TGF-β pathways [117]. Studies have shown that CAFs enhance the migration (through CCL2 and CCL5 secretion) and invasion (through CCL7 and CXCL16) of HCC cells in vitro and could promote HCC metastasis to distant sites, including the bone.

Once in the bone marrow, disseminated cancer cells that survive the long journey can enter metastatic dormancy as individual cells or can be found as small, non-expanding masses. This helps the tumor cells adapt to the bone microenvironment and evade immune surveillance [20]. Reactivation and outgrowth of these dormant tumor cells, which share properties with stem cells, occur in response to niche signals after a lag time that may last months or years. Hematopoietic progenitor cells recruited in the niche also remodel the extracellular matrix by secreting MMP9, for example, and promote the formation of angiogenic sprouts to sustain cancer cell reactivation and proliferation [118]. In the bone, Ki67^+^ vasculature sprouts secrete biomolecules, like periostin, fibronectin, tenascin, versican, and transforming growth factor-β1 (TGF-β1), to support the growth of cancer cells located in close proximity [119]. The hypoxic environment of the bone cavity enhances the reactivation process. The growth factors, cytokines, and bone-resorbing molecule-enriched bone matrix also lead to the exit of cancer cells from the dormancy state [119].

Due to the low incidence of bone metastasis in patients with HCC and CCA, optimal treatment strategies remain unclear. Patients with hepatobiliary cancers who develop bone metastases are typically considered for systemic therapies alongside palliative care and bone-targeted localized treatments. Complications related to bone metastasis, such as severe pain, spinal cord compression, pathological fractures, and mobility impairments, significantly affect quality of life. Currently, there are no widely accepted screening protocols for detecting and treating bone metastasis in hepatobiliary cancer patients. Therefore, efforts to identify molecular and cellular pathways that could potentially be targeted to mitigate bone metastasis from hepatobiliary cancers are essential. MiRNAs and RBPs are potential candidate molecules, as discussed below.

### 4.1. miRNAs in Promoting Bone Metastasis: Insights from Preclinical Models

miRNAs can influence cells in the bone microenvironment to enhance their ability to support metastatic tumor cells. Although the role of miRNAs in bone metastasis is well understood, few studies have explored this in the context of hepatobiliary tumors. In an effort to investigate whether the lncRNA zinc finger E-box binding homeobox 1 antisense 1 (ZEB1-AS1) could control bone metastasis in HCC cells, Ma et al. found that lncZEB1-AS1 was highly expressed in patients with HCC with extrahepatic metastases compared to those without metastases [120]. The expression of lncZEB1-AS1 was linked to bone metastasis and poor outcomes in a large group of HCC patients compared to the controls. Additionally, the authors found that the targeting of miR-302b by this lncRNA resulted in increased *EGFR* expression and activation of the EGF-mediated PI3K–AKT pathway, leading to the expression of metalloproteases (MMP), such as *MMP-2*, *-7*, and *-9* [120]. Therefore, targeting the lncZEB1-AS1–miR-302b–EGFR axis could be a potential strategy to prevent bone metastasis in HCC patients.

Another study showed that Lnc34a was highly expressed in HCC and correlated with bone metastasis [20]. The pro-bone metastatic effect of Lnc34a was achieved through its epigenetic influence on miR34a by recruiting DNMT3a through PHB2 to methylate the miR-34a promoter, resulting in the silencing of this miRNA. This suppression hindered its impact on TGF-β1-target gene expression in hepatoma cells. Interestingly, MRX34, a miR-34 mimic encapsulated in liposomes, was utilized in a groundbreaking Phase 1 study, which determined a recommended Phase 2 dose of 70 mg/m^2^ for HCC [121]. Effective delivery of this liposomal formulation to solid tumors was observed, along with a favorable safety profile and notable inhibition of target genes in white blood cells. The usage of miR34-mimics in CCA warrants further investigation due to conflicting reports on the role of miR-34 in this type of tumor [122,123].

Studies describing the involvement of miRNAs in HCC metastasis to distant organs, other than the bone, in preclinical models may provide insights into dissecting the role of this non-coding RNA in invasion and colonization of the bone by circulating hepatobiliary cells [124]. The participation of miRNAs in the progression of CCA has also been documented [125]. For instance, miR-21 overexpression in CCA was associated with reduced expression of programmed cell death 4 (*PDCD4*) and the tissue inhibitor of MMP-3 (*TIMP-3*), showing oncogenic activity [126]. miR-21 was found to regulate PTEN in CCA cell lines [127]. miR-191 also showed upregulation in iCCA versus normal bile duct tissues [75]. Importantly, overexpression of this miRNA induced proliferation, invasion, and migration of CCA cells in vitro in QBC939 and HuCCT1 cells and in vivo in tumors generated by QBC939 cells modulated for miR-191 expression in rodents. In this model, lung metastasis of miR-191-overexpressing cells was observed. On the other hand, miR-7-5p was found downregulated in iCCA tissues and cell lines compared to normal controls, and this down-regulation of miR-7-5p expression was associated with poor outcomes of iCCA patients [128]. miR-7-5p targets myeloid differentiation factor 88 (MyD88) to dampen tumor cell proliferation, migration, and invasion of iCCA cells in vitro, thus acting as a tumor suppressor.

Low miR-122 expression is frequently observed in HCC and CCA, and this is associated with larger tumors, metastasis, and poor prognosis [129]. miR-122 targets several oncogenes, such as *c-Myc*, as well as genes involved in tumor-promoting processes, like EMT and angiogenesis. Therefore, preclinical studies suggest that restoring therapeutic levels of miR-122 can be beneficial for HCC and CCA patients [63,130]. Zhang et al. used a combination of chemotherapy and miRNA to create nanoparticles encapsulated with gemcitabine and oleic acid prodrugs, along with miR-122 [131]. These functionalized nanoparticles efficiently homed to tumors in nude mice and inhibited HCC growth in vivo. They also demonstrated a good biosafety profile, showing promise for further evaluation for potential clinical translation.

Reports on the role of miRNAs in osteotropism, the formation of pre-metastatic and metastatic niches, and dysregulated bone remodeling in other types of cancers can help uncover the molecular mechanisms behind bone metastasis development from hepatobiliary cancers [132]. Extracellular vesicles (EVs), which are rich in miRNAs, play a crucial role in preparing the bone microenvironment for tumor cell homing. For example, EVs released by prostate cancer stem cells (CSCs) carry high levels of miR-183, which promotes osteoclast formation and facilitates the creation of metastatic niches by enabling these cells to degrade the extracellular matrix [132]. Other studies have identified additional cancer-derived EVs containing miRNAs that influence osteoclast differentiation, such as miR-152–3p, miR-378a-3p, miR-214-3p, and miR-325-3p [133]. EVs also have the potential to serve as anti-tumor therapeutic agents. For instance, Li et al. demonstrated that EVs derived from a hepatic stellate cell line (LX-2) and enriched in miR-195 could inhibit the proliferation, migration, and metastasis of HuCCT-1 CCA cells in vitro [134]. Furthermore, intravenous injection of miR-195-loaded EVs inhibited iCCA tumor growth and extended survival in a rat model of CCA.

However, miRNAs may have multiple targets, and their dual role in the evolution of hepatobiliary cancers and distant metastasis further complicates this issue. For instance, it was shown that Let-7c expression was downregulated in CCA compared to normal adjacent tissues. It could inhibit migration and invasiveness of CCA cells in vitro but promoted distant metastasis in vivo [135]. Therefore, caution should be exercised when extrapolating miRNA-based data from one cancer type to another and from primary tumors to metastatic tumors.

### 4.2. RBPs in Modulating Bone Metastasis: Insights from Other Cancers

RBPs can fine-tune processes involved in tumor cell survival, invasion, and metastatic spread. Several recent studies point to a role played by RBPs in bone pathophysiology. For example, Musashi 2 (MSI2), the expression of which is induced by the receptor activator of the NF-κB ligand (RANKL) during osteoclast differentiation, can promote osteoclastogenesis by inducing RANKL-induced nuclear factor-κB (NF-κB) to prevent osteoclast apoptosis [136]. Other RBPs are also involved in bone biology and have been recently described elsewhere [137].

EMT and its reverse process mesenchymal–epithelial transition (MET) are steps of metastasis that can be regulated by RBPs. ESRP1 and ESRP2 are two RBPs with well-known functions in these processes. Downregulation of ESRP expression induces the alternative splicing of transcripts involved in EMT [138]. Conversely, we have demonstrated that overexpression of ESRP1 can also promote EMT, tumor cell proliferation, anchorage-independent growth of human colorectal cancer cells in vitro, and macrometastasis in experimental metastatic models in vivo. This is achieved by inducing the expression of SNAI1 through the FGF7/FGFR2IIIb/PI3K/Akt pathway [139]. Moreover, overexpression of ESRP1 induces the expression of the tumorigenic variant *RAC1B* of *RAC1* in these cells [140]. Another example of an RBP involved in EMT is the A-kinase anchor protein (AKAP8) [141]. It has been shown that AKAP8 can inhibit the activity of the splicing factor and EMT-promoter heterogeneous nuclear ribonucleoprotein M (hnRNPM), thus inhibiting EMT and breast cancer metastasis in murine models. Additionally, AKAP8-induced calsyntenin 1 (*CLSTN1*)-S-splicing isoform formation promotes epitheliality in breast cancer cells. On the other hand, a recent study demonstrated that AKAP8 is overexpressed in ovarian cancer and promotes cancer progression and metastasis by modulating the formation of the alternative transcript hnRNPUL1-S [142]. This alternative transcript blocks the cytotoxicity of poly (ADP-ribose) polymerase (PARP) inhibitors in ovarian cancer and is involved in ovarian cancer cell proliferation, invasion, and migration. Like miRNAs, RBPs can play dual roles in the multistep processes of cancer metastasis, highlighting the need for a detailed study of tumor types before addressing therapeutic targeting of these biomolecules involved in RNA metabolism.

Bone metastasis is significantly influenced by RBPs, and the role of RBP-related alternative splicing events in human health and disease has recently been extensively reviewed in [143]. For example, RBM3, a cold-stress response RBP highly expressed in cancer cells, promotes N6-methyladenosine (m^6^A) modification of mRNA. In prostate cancer, RBM3 has been shown to impact the alternative splicing of *CD44* [144]. Specifically, overexpression of RBM3 reduces the expression of *CD44v8-v10* and promotes the splicing of *CD44s*, which is known to have an inhibitory effect on metastasis, thus reducing the stemness properties of prostate cancer cells (PC3 cells). RBM3 also modifies the 3′ untranslated region (UTR) of catenin beta 1 (*CTNNB1*), leading to decreased mRNA stability [145]. This decrease in β-catenin protein levels results in a reduction in Wntβ-catenin signaling, ultimately impacting interactions between osteoblasts and the maintenance of prostate cancer cell stemness [145]. Since RBM3 expression is suppressed in the bone microenvironment, a potential therapeutic strategy could involve restoring RBM3 expression to inhibit bone metastasis in prostate cancer.

Human antigen R (HuR), another RBP, enhances mRNA stability containing AU-rich elements by binding to the 3′UTR, thereby increasing their translation [146]. High expression of HuR has been reported in several cancers, including HCC, where HuR expression has been associated with poor prognosis in patients [147]. The HuR protein interacts with the 3′UTR of methionine adenosyltransferase (MAT)-2A mRNA, which, along with MAT1A, regulates the production of S-adenosylmethionine (SAM)—a critical molecule for hepatocyte proliferation and differentiation [147]. Elevated MAT2A expression has been linked to HCC cell de-differentiation and accelerated proliferation. HuR expression is significantly elevated in human HCC. Additionally, high cytoplasmic HuR expression has been independently associated with the effectiveness of adjuvant gemcitabine-based chemotherapy in patients with resected CCA [148]. However, the relationship between HuR expression and bone metastasis from hepatobiliary cancers has not yet been investigated. Insights from a study on breast cancer bone metastasis suggest that in a mouse model of experimental metastasis, HuR knockdown significantly inhibited MDA-MB-231 cell-induced tumor growth in the tibial bone marrow and osteolysis [149]. CC chemokine ligand 20 (CCL20), regulated by HuR and secreted into the bone microenvironment, can influence the production of RANKL, which is involved in the differentiation and activity of osteoclasts, thereby affecting bone metastasis. These findings suggest that *CCL20*, a target of HuR, may become a potential therapeutic target for patients with bone metastasis from breast cancer.

Recently, the ribosomal protein 7 (RPS7) was found to be upregulated in HCC tissues compared to controls [89]. Preclinical studies on human HCC cell lines (MHCC97H and HLE) and in orthotopic or experimental metastasis HCC mouse models showed that CRISPR/Cas9-mediated knockout of RPS7 led to reduced growth, migration, and invasion of tumor cells in vitro. In vivo, RPS7 knockout resulted in reduced tumor growth in the liver of the orthotopic model and fewer metastatic nodules in the experimental metastasis model. Interestingly, RPS7 regulates the expression of *LOXL2* at the post-transcriptional level and activates the LOXL2-mediated ITGB1/FAK/SRC signaling pathway to promote metastasis. Therefore, designing strategies to block this RPS7-induced metastatic pathway may lead to the discovery of molecular drugs against HCC and metastasis.

RNA-binding motif 43 (RBM43) acts as a tumor suppressor with significantly low expression in HCC and even lower expression in metastatic tumors [91]. CRISPR/Cas9-generated Rbm43-deficient HepG2 and QGY-7703 cells showed enhanced migration and invasion abilities, which were reversed upon restoration of RBM43 expression. The absence of RBM43 led to increased mRNA stability and expression of Slug, a factor known to promote stemness, invasion, migration, and metastasis in HCC [150]. Therefore, the RBM43 is another metastasis suppressor that is worth targeting in HCC to prevent metastatic spread to distant sites, such as the bone.

The anillin actin-binding protein (ANLN) is highly expressed in patients with HCC and bone metastasis [151]. It was found that m^6^A modifications, carried out by methyltransferase 3, the N6-adenosine-methyltransferase complex catalytic subunit (METTL3), and the canonical RBP, YTH N6-methyladenosine RNA binding protein F1 (YTHDF1), stabilize ANLN mRNA. Increased nuclear ANLN levels can trigger RANKL expression through the SP1/KIF2C/mTORC pathway, causing an imbalance in RANKL:OPG expression in the bone microenvironment. This imbalance promotes tumor cell invasion of the bone, emphasizing the potential of targeting other complexes modulated by RBPs as therapeutic strategies for bone metastasis.

Although RBPs are considered challenging therapeutic targets, attempts to develop MSI-specific inhibitors have shown promise, especially in preclinical studies. RNA interference-based strategies for inhibiting these proteins have yielded encouraging results [152]. For instance, MSI1 and MSI2 were found to be potentially “druggable” by antisense oligonucleotides in a human pancreatic cancer line and a pancreatic cancer mouse model [153]. Of particular interest was the fact that targeting MSI1 expression inhibited pancreatic cancer growth in a human cell line and in a patient-derived xenograft in vivo. This approach may also be used for MSI2, which is highly expressed in HCC and CCA and is representative of enhanced tumor growth, distant metastasis, resistance to chemotherapy, and poor prognosis [154]. Interestingly, it was shown that MSI2 short hairpin RNA (shRNA)-mediated knockdown dampened CCA cell (QBC939 cell line) growth, migration, and invasion in vitro [155].

## 5. Future Therapeutic Perspectives and Challenges in Bone Metastasis

Patients with bone metastasis typically undergo surgical resection when possible, often accompanied by chemotherapy and/or radiotherapy, taking into account the patients’ overall health, cancer type, and presence of metastases outside the bone [156]. However, once tumor cells establish in the bone, the disease is rarely curable. Therefore, it is essential to develop therapeutic strategies that can predict and overcome resistance to chemotherapy, as well as inhibit metastatic spread. Preclinical studies utilizing in vivo models of human cancers that metastasize to the bone, advanced molecular imaging systems, generation of patient cell-derived organoids in vitro, and computational oncology have proven to be invaluable in this endeavor. Some of these approaches were used to create an in silico model capable of predicting the response of prostate cancer to a bone-targeting isotope, ^223^Radium. This isotope has been shown to shrink tumors and extend the overall survival of cancer patients by 3.6 months compared to those treated with a placebo [157]. This study, based on in vivo data and computational techniques, illustrated how ^223^Radium accumulated at the bone interface, affecting the amount of drugs delivered to the tumor and therapeutic efficacy based on distance. Microlesions closer to the bone interface received therapeutic radiation doses compared to those located more than 400 μm away. These model systems are also beneficial in avoiding unnecessary radiation in patients who would not benefit from this treatment.

The next frontier in bone metastasis treatment is cellular immunotherapy. A preclinical study conducted on prostate cancer metastasizing to the bone revealed that the tumor cells express ligands (ULBP1, MICA, and MICB) capable of activating γδ TCR-bearing T cells, as well as the prostate stem cell antigen (PSCA) [158]. Building on this knowledge, Frieling et al. developed PSCA-targeting γδ chimeric antigen receptor-modified T (CAR-T) cells, which decreased the viability of prostate cancer cells and reduced the tumor size in vivo in a mouse model of intratibial prostate tumors [158]. The rate of tumor regression was enhanced with the co-administration of zoledronate (ZOL), a bisphosphonate used to maintain bone density. Importantly, treatment of the prostate cancer mice with anti-PSCA γδ CAR-T cells reduced osteolytic lesions by accumulating in the tumor-containing bone, demonstrating the effectiveness of this treatment in eliminating or significantly reducing bone tumor burden. The safety of CAR-T cell-based therapy was also demonstrated in a Phase I clinical trial (clinicaltrials.gov ID: NCT01869166) in CCA (biliary tract cancer) patients with unresectable, relapsed/metastatic EGFR-positive tumors [159]. Some refinements are necessary before clinical translation can be pursued, especially with regard to their brief permanence (around 1 month) in tumor tissues [158].

As ongoing clinical trials continue to investigate the potential of immune checkpoint blockade and traditional Chinese medicines in the therapeutic venues for HCC and CCA, stem-cell-based therapy and immunotherapy may also bring a notable transformation in this treatment landscape due to their multi-faceted mechanisms in combating these tumors [160,161,162]. An interventional Phase1/2 clinical study (clinicaltrials.gov; ID: NCT02089919), investigated the possibility of generating vaccines targeting CSCs by injecting autologous dendritic cells (DCs) pulsed with the whole extracts of aldehyde dehydrogenase (ALDH)^high^ or ALDH^low^ tumor cells in HCC patients, based on their previous results in murine models [163]. No results have been posted for this completed clinical study, but the preclinical work demonstrated that CSCs were highly immunogenic, and that DCs primed with ALDH^high^ CSC lysates could efficiently reduce tumor growth and lung metastasis. Other clinical studies on DC-vaccine-based immunotherapy, as monotherapy or in combination with different anticancer therapies for HCC, have been reported and recently described in [164]. Moreover, at the preclinical level, several important questions have been addressed to optimize DC-based anti-tumor vaccines, including which strategies to employ for tumor antigen priming and cell maturation, as well as the use of nanoparticles and EVs [164]. With regard to the latter, it has been shown that EVs obtained from HCC cells carry HCC antigens that can efficiently activate DCs in vitro, while primed DC-derived EVs can enhance the anti-cancer immune responses against HCC by participating in CD8^+^ T cells’ activation and by remodeling the tumor microenvironment in animal models [164,165,166,167]. DC vaccines have also shown promise for CCA, especially in combination with other therapies [168]. A study on CCA patients reported that DC-based immunotherapy against the Wilms tumor 1 and Mucin 1 synthetic peptides could improve the mean CCA patients’ survival time [169]. Autologous tumor lysate-pulsed DC-generated vaccine plus ex vivo-activated T-cell transfer in iCCA patients led to significantly enhanced progression-free and overall survival (18.3 and 31.9 months, respectively) with respect to those who underwent surgical resection alone (7.7 and 17.4 months, respectively) [170]. The effects of these treatments on the development of bone metastasis still need to be explored.

Regarding miRNAs and RBPs that are potential therapeutic targets in HCC- and CCA-mediated bone metastasis, improvement in drug delivery systems and carriers is urgently needed, as recently reported [156]. Combining suitable nanocarriers, including inorganic-, polymer-, and lipid-nanoparticles, EVs, and liposomes, with miRNA- and RBP-based therapeutics, to prevent bone metastasis is still in the early stages [171]. Several miRNAs have been found to be associated with drug resistance in HCC, particularly those involved in apoptosis, autophagy, cell proliferation, EMT, or control of transmembrane drug transporters of the multidrug resistance gene family, demonstrating involvement in resistance to anthracyclines, cisplatin, and sorafenib therapies [172]. For example, miR-222 or -494, which target PTEN, participated in resistance to sorafenib in HCC cells. On the other hand, miR-451a (downregulated in HCC and CCA tissues and in serum EVs of biliary tract cancer patients) can block the advancement of CCA and GC (biliary tract cancers) resistant to gemcitabine by suppressing the activation of the PI3K/AKT pathway induced by the migration inhibitory factor (MIF), a survival-promoting factor [173]. Two main strategies are being employed to target miRNAs, which aim at either replacing, restoring, overexpressing, downregulating, reducing, or inhibiting the miRNAs of interest in cancer, known as miRNA mimics or antagomiRs, respectively [174]. Examples of miRNA mimics or antagomiRs employed in HCC are listed in Table 3.

Importantly, transfection of miR-451a mimics in CCA and GC cancer cells eliminated these effects. Therefore, significant effort is being directed towards developing delivery strategies, both vector-mediated and vector-free, to target oncomiRs or deliver anti-neoplastic miRNAs directly to tumor cells without impacting adjacent or normal tissues. These strategies aim to achieve good cellular uptake, high stability, minimal immune response, and low cytotoxicity. Targeting RBPs by designing specific strategies against their unique domains that facilitate binding with RNA or other RBPs could evolve into a therapeutic approach to prevent bone metastasis from primary tumors. Due to the pleiotropic effects of RBPs on cellular homeostasis, blocking oncogenic RBP function exclusively in tumor cells, possibly through functionalized liposome-mediated delivery, may offer a solution.

Other strategies include the use of small molecule inhibitors capable of blocking the RNA–RBP interaction, upstream kinase activity, RBP post-transcriptional modifications, or enzymatic activity, as well as inducing RBP degradation. Peptides that inhibit RBP interaction with other functional proteins, antisense technology, such as antisense oligonucleotides (ASOs) that bind to mRNAs and mediate RNase H-mediated degradation, splice-switching oligonucleotides (SSOs) that block RNA-RNA base-pairing or protein-RNA binding to disrupt normal splicing activity, and siRNAs or aptamers that can lead to the degradation of RBP mRNA or prevent RBP–RNA interaction, are only some potential options [181]. Circular RNAs and CRISPR/Cas9 technology are also promising tools that need exploration as cancer RBP therapeutics. Further investigations are needed regarding the metastatic invasion of the bone by hepatobiliary cancer cells concerning miRNAs and RBPs, considering their prime and interacting but possible dual roles in the metastatic cascade.

## 6. Conclusions

The complexity and aggressiveness of HCC and CCA, along with the development of chemoresistance, present significant challenges in the development of effective therapeutics. This leads to an increase in metastasis, including to distant sites, such as the bone. Molecules involved in RNA metabolism, such as miRNAs and RBPs, play a role in various tumorigenic processes, from primary tumor formation to metastatic growth. Their dysregulated expression or activity in hepatobiliary cancers can serve as diagnostic, prognostic, and chemotherapy resistance-predicting biomarkers. Future research should investigate the regulation of oncogenic miRNAs and RBPs expression in depth, while considering their complex interaction network and activities in hepatobiliary cancers and related metastasis in order to design new treatment perspectives for these deadly human diseases.

## Figures and Tables

**Table 1:** Examples of miRNAs and targets showing dysregulated expression in HCC and CCA ^1^.

miRNA	Type of Tumor	Role/Mechanism	Expression in Tumor Tissue (vs. Control)	Reference
miR-122	HCC with intrahepatic metastasis	Tumor suppressor: reduces cell proliferation, migration, and invasion of tumor cells and regulates local invasion in vivo.	↓	[61]
	CCA	Tumor suppressor: reduces cell proliferation and invasion and stimulates apoptosis by controlling p53 expression.	↓	[62,63]
miR-29	HCC	Tumor suppressor: regulates proliferation, neoangiogenesis, and metastasis by targeting *IGF2BP1*, *VEGFA*, and *BCL2*. MiR-29c-3p targets *TPX2* involved in chromosomal instability. miR-29a targets *STAT3*, thus inhibiting cell proliferation, migration, and invasion.	↓	[58,64,65,66]
	CCA	Tumor suppressor: prevents *CDKN2B* promoter methylation by targeting DNMT3B.	↓	[67]
miR-195	HCC	Tumor suppressor: inhibits the expression of *VEGF*, *VAV2*, and *CDC42*.	↓	[68]
miR-101	HCC	Tumor suppressor: inhibits cell proliferation, migration, and invasion though Girdin inhibition.	↓	[69]
	CCA	Tumor suppressor: inhibits angiogenesis by targeting VEGF.	↓	[70]
miR-141	HCC	Tumor suppressor: targets *STAT4* expression to control liver cancer cell proliferation, migration, and invasion, as well as the metastasis-promoting gene *Tiam1*.	↓	[60,71]
	CCA	Oncomir: exosomes miR-141 may induce gemcitabine resistance in iCCA.	↑	[72,73]
miR-191	HCC	Oncomir: promotes cell proliferation by targeting the has_circ_0000204/miR-191/KLF6 axis.	↑	[74]
	iCCA	Oncomir: targets the TET1–p53 pathway.	↑	[75]
miR-181b-5p	CCA	Oncomir: targets *PARK2* via the PTEN/PI3K/AKT pathway to promote cell proliferation, migration, and invasion.	↑	[76]
miR-129-2	HCC	Tumor suppressor: inhibits *HMGB1* to suppress cell migration and invasion.	↓	[77]
miR-200b	HCC	Tumor suppressor: targets *ERG*, *ZEB1/2*, and *Notch1* to inhibit invasion and migration of cancer cells.	↓	[78]

^1^ This table summarizes reported roles and mechanisms of action of miRNAs that either act as oncogenes or tumor suppressors in HCC or CCA. Abbreviations: BCL2, B-cell lymphoma 2; CCA, cholangiocarcinoma; CDC42, cell division cycle 42; CDKN2B, cyclin-dependent kinase inhibitor 2B; CLOCK, clock circadian regulator; DNMT3B, DNA methyltransferase 3B; ERG, ETS-related gene; HCC, hepatocellular carcinoma; HMGB1, high-mobility group box 1; IGF2BP1, insulin-like growth factor 2 mRNA-binding protein 1; KLF6, KLF transcription factor 6; mTOR, mammalian target of rapamycin; PI3K, phosphatidylinositol 3-kinase; PTEN, phosphatase and tensin homolog; PTPN12, protein tyrosine phosphatase non-receptor type 12; STAT, signal transducer and activator of transcription; TET1, ten-eleven translocation 1; Tiam1, T lymphoma invasion and metastasis 1; TPX2, targeting protein for Xklp2; VAV2, Vav guanine nucleotide exchange factor 2; VEGFA, vascular endothelial growth factor A; Zeb1/2, zinc finger E-box binding homeobox 1/2; ↓, expression of gene is upregulated in tumor tissue; ↑, expression of gene is downregulated in tumor tissue.

**Table 2:** Examples of dysregulated expression of RBPs in hepatobiliary cancers ^1^.

RBP	Type of Tumor	Role/Mechanism	Expression in Tumor Tissue (Versus Control)	Reference
MSI1 and MSI2	HCC	Oncogenes: enhance HCC invasion by inducing EMT.	↑	[84]
Nucleolin	iCCA	Oncogene: enhances CCA proliferation, growth, and invasion upon lactylation by the acyltransferase p300 at lysine 477, increasing MADD translation and ERK activation.	N/A	[85]
SRSF1	HCC	Oncogene: enhances cell proliferation, survival, and tumorigenesis through alternative splicing of the oncogenic isoforms of the antiapoptotic gene *BIM* and the oncogenes *S6K1* and *TEAD1* when its expression is induced by the lnc-RNA MALAT1.	↑	[86]
hnRNP A1	HCC	Oncogene: enhances the expression of *CD44v6*, increasing HCC invasiveness and resulting in a poor prognosis for HCC patients after curative resection.	↑	[87]
RPS5	HCC	Oncogene: regulates the cell cycle and metastasis.	↑	[88]
RPS7	HCC	Oncogene: promotes HCC cell adhesion, migration, invasion, and lung metastasis.	↑	[89]
RBM39	CCA	Oncogene: promotes CCA cell growth through the EZH2/WNT7B/β-catenin pathway.	↑	[90]
RBM43	HCC	Tumor suppressor: targets *Slug* mRNA stability and expression in HCC.	↓	[91]
CCDC137	HCC	Oncogene: modulates the subcellular localization of its target mRNAs by binding to the microprocessor complex subunit, DGCR8, and activates AKT signaling.	↑	[92]
PIWIL4	iCCA	Oncogene: activates the mTOR signaling pathway.	↑	[93]
SUPT5H	iCCA	Oncogene: involved in tumor cell proliferation, migration, the cell cycle, and apoptosis.	↑	[93]
SORBS2	HCC	Tumor suppressor: stabilizes *RORA* mRNA by binding to its 3′UTR, hence promoting its expression, which is involved in the suppression of tumorigenesis and metastasis.	↓	[94]

^1^ This table summarizes reported roles and mechanisms of RBPs that either act as oncogenes or tumor suppressors in HCC or CCA. Abbreviations: DGCR8, DiGeorge syndrome critical region 8; EMT, epithelial–mesenchymal transition; hnRNP A1, heterogeneous nuclear ribonucleoprotein A1; lnc-RNA, long non-coding RNA; MADD, MAP kinase-activating death domain protein; MSI1/2, Musashi 1/2; PIWIL4, PIWI-like RNA-mediated gene silencing 4; RBM, RNA-binding motif protein; RORA, RAR-related orphan receptor A; RPS5, 40S ribosomal protein S5; RPS7, ribosomal protein S7; SRSF1, serine- and arginine-rich splicing factor 1; SORBS2, sorbin and SH3 domain-containing 2; SUPT5H, SPT5 homolog, DSIF elongation factor subunit; EZH2, enhancer of zeste homolog 2; ↓, expression of gene is upregulated in tumor tissue; ↑, expression of gene is downregulated in tumor tissue.

**Table 3:** Examples of potential miRNA- and RBP-based therapeutics for HCC ^1^.

miRNA	Type of Intervention	Vector	Reference
miR-342-3p	Overexpression	Adeno-associated virus vector	[175]
miR-34	Replacement	Liposome (MRX34)	[121]
miR-122	Inhibition	Locked nucleic-acid-modified DNA phosphorothioate antisense oligonucleotide (LNA, miravirsen or SPC3649)	[176]
miR-21	Inhibition	Small molecule (AC1MMYR2)	[177]
miR-335-5p	Delivery/restoration	LX2-derived exosomes	[178]
**RBP**	**Type of intervention**	**Approach**	**Reference**
RBM3	Target inhibition (YAP1)	Lentivirus-mediated YAP1-silencing	[179]
Cold-inducible RNA-binding protein (CIRP)	Vaccination	CIRP-containing immunogens in combination with immune checkpoint inhibitors	[180]

^1^ Most miRNA-based therapies are currently being tested for their long-term effects after injection in humans, while the optimal methods for modulating RBP expression are still under investigation.

## Data Availability

No new data were created or analyzed in this study. Data sharing is not applicable to this article.
